# The role of spatial information in an approximate cross-modal number matching task

**DOI:** 10.3758/s13414-023-02658-9

**Published:** 2023-01-31

**Authors:** Marco Carlo Ziegler, Luisa Karoline Stricker, Knut Drewing

**Affiliations:** 1 Department of Psychological Assessment, Justus-Liebig-University Giessen, Giessen, Germany; 2 Department of Experimental Psychology - HapLab, Justus-Liebig-University Giessen, Giessen, Germany

**Keywords:** Haptics, Multisensory processing, Visual perception

## Abstract

The approximate number system (ANS) is thought to be an innate cognitive system that allows humans to perceive numbers (>4) in a fuzzy manner. One assumption of the ANS is that numerosity is represented amodally due to a mechanism, which filters out nonnumerical information from stimulus material. However, some studies show that nonnumerical information (e.g., spatial parameters) influence the numerosity percept as well. Here, we investigated whether there is a cross-modal transfer of spatial information between the haptic and visual modality in an approximate cross-modal number matching task. We presented different arrays of dowels (haptic stimuli) to 50 undergraduates and asked them to compare haptically perceived numerosity to two visually presented dot arrays. Participants chose which visually presented array matched the numerosity of the haptic stimulus. The distractor varied in number and displayed a random pattern, whereas the matching (target) dot array was either spatially identical or spatially randomized (to the haptic stimulus). We hypothesized that if a “numerosity” percept is based solely on number, neither spatially identical nor spatial congruence between the haptic and the visual target arrays would affect the accuracy in the task. However, results show significant processing advantages for targets with spatially identical patterns and, furthermore, that spatial congruency between haptic source and visual target facilitates performance. Our results show that spatial information was extracted from the haptic stimuli and influenced participants’ responses, which challenges the assumption that numerosity is represented in a truly abstract manner by filtering out any other stimulus features.

## Introduction

The ability to process discrete quantity can be demonstrated in humans, primates, and other animal species (Bisazza & Gatto, 2021; Brannon & Merritt, 2011; Butterworth, 2010; Dehaene, 2011; Feigenson et al., 2004). A cognitive system, the approximate number system (ANS), is thought to be an innate and evolutionary ancient part of number processing, which is shared among humans and other animals (Brannon & Merritt, 2011; Butterworth, 2010; Dehaene, 2011; Nieder, 2016; Spelke & Kinzler, 2007). The ability to quickly estimate numerosity was most likely advantageous for individual fitness (Lourenco & Aulet, 2022). A rapid estimate of numerosity, for example, may have helped to identify which herd has more prey and ultimately deciding which herd to hunt down. Also in modern human life, the ability to accurately estimate numerosity is beneficial every day. It helps one for example to increase the chance of getting a seat in a train during rush hour (or get into the train at all), since accurately estimating the number of people waiting for an arriving train on different train sections allows one to predict the fastest way into the train. The ANS is assumed to enable a fuzzy representation of number without counting (Park & Brannon, 2013). The ANS is reported to process items, objects, or events >4 (Feigenson et al., 2004; Mou & vanMarle, 2014; Olsson et al., 2016; Park & Brannon, 2013; Spelke & Kinzler, 2007).

Three signatures are commonly attributed to the ANS: ratio dependency, nonsymbolic arithmetic operating, and amodality (Brannon & Merritt, 2011). Ratio-dependency is demonstrated when individuals compare which of two numerosities is the larger (e.g., when comparing two dot arrays). The difficulty of the comparison is determined by the ratio of the two numbers rather than their absolute value (e.g., a 10 to 5 comparison is equally difficult to a 20 to 10 comparison). Subsequently, a 5 to 6 comparison is easier than a 7 to 8 comparison even though the absolute difference is 1. Nonsymbolic arithmetic operating is a form of calculation with abstract distinct quantity (e.g., the addition of two sets of dot patterns), which can be performed by children even before formal mathematical education (Barth et al., 2006; Gilmore et al., 2010). The third signature, amodality, is a widely assumed axiom based on the idea that the ANS extracts numerosity as an abstract feature from any suitable stimulus material, thereby assuming that the representation of number is amodal (Brannon & Merritt, 2011; Dehaene, 2011; Dehaene & Changeux, 1993; Gebuis et al., 2016; Tokita & Ishiguchi, 2016).

The ANS theory describes a process in different stages of how the percept of numerosity is shaped (Dehaene, 2011; Dehaene & Changeux, 1993; Gebuis et al., 2016). In the first “sensory” stage, the source stimulus is processed by the receiving modality. In vision, this can be a set of dot arrays in varying sizes and positions. In the second stage, the normalizing phase, all stimulus properties are removed and only a standardized signal for each dot remains. In stage three, the accumulation phase, the standardized signals, as well as some degree of error, are summed up into the final percept of numerosity (Gebuis et al., 2016; cf. Tokita et al., 2013). This understanding of the ANS is sometimes referred to as a “direct ANS model” (Qu et al., 2022). The direct ANS model of numerosity estimation often seems to serve as a default theory in the field of numerical cognition (Dehaene, 2011; Halberda et al., 2008; Hyde, 2011; Spelke & Kinzler, 2007). Competing, but to our experience less prevalent in literature, are “indirect models,” which assume that a numerosity percept arises from perceiving indirect surrogate cues (e.g., the spatial distribution of stimulus pattern; Qu et al., 2022). Only recently, alternative perspectives or extensions of the direct model have been proposed (Allïk & Tuulmets, 1991; Clarke & Beck, 2021; Gebuis et al., 2016; Gevers et al., 2016; Leibovich, Katzin, Harel, & Henik, 2017a; Lourenco & Aulet, 2022; Walsh, 2003; Zorzi & Testolin, 2018). These perspectives differ in their general view of whether number is a distinct feature and privileged entity in perception or a construct of surrogate perceptional cues (Lourenco & Aulet, 2022; Qu et al., 2022). Furthermore, they distinguish themselves in the question whether the “number sense” is innate (Spelke & Kinzler, 2007), emerges from general, not domain specific abilities, based on exposition and interaction with the environment (Zorzi & Testolin, 2018) or is nonexistent at all (Gebuis et al., 2016). Gebuis et al. (2016), for example, propose that numerosity estimation is the result of integrating different sensory cues, such as distance between stimuli or their convex hull, which only shape a numerosity estimate as required. Other authors argue for a model that considers both number and other magnitudes “holistically” to shape a magnitude percept (Leibovich, Katzin, Harel, & Henik, 2017a). Recently, Lourenco and Aulet (2022) summarized that nonnumerical features such as space and area are sustained throughout processing as magnitude information encoded along with number; the experience of number is an attention-based “reading out” of an integral representation. However, the scope of this proposal is explicitly limited to the visual number processing (Lourenco & Aulet, 2022). This raises the question of whether and how the stream of numerical and nonnumerical information functions throughout sensory and cognitive processing when the source modality is any other than the visual. In summary, some of the proposals and theories have sparked some controversial discourse (Clarke & Beck, 2021; Leibovich, Katzin, Salti, & Henik, 2017b).

Both model types, the direct model and indirect models, can explain phenomena of approximate number processing (e.g., ratio dependency). However, the traditional direct ANS framework conflicts with accumulating evidence, which demonstrates that nonnumerical information (e.g., the spatial area covered by nonsymbolic stimuli or their spatial arrangement) systematically affects the numerosity percept as well (Clayton et al., 2015; DeWind et al., 2015; Gilmore et al., 2016; Hendryckx et al., 2021; Szucs et al., 2013; Tomlinson et al., 2020). DeWind et al. (2015) found that factors that influence the approximate numerosity perception can be formalized into a taxonomy of number, area, and space. They presented a method to statistically quantify these factors and empirically demonstrated that besides number the features space and area additionally contribute to a numerosity percept in a visual dot comparison task (DeWind et al., 2015; DeWind & Brannon, 2016). A consistent finding is that participants use spatial cues (factor space), such as field area, convex hull, or sparsity of a dot array, when comparing two dot arrays (Clayton et al., 2015; Gilmore et al., 2016; Hendryckx et al., 2021). Bertamini et al. (2016) ran an experiment to explicitly identify effects of the spatial arrangement of dot patterns, such as local clustering and occupancy area, on the numerosity and related percepts. They presented participants visual dot arrays in different spatial configurations while keeping the number of dots constant. Indeed, spatial configuration, including local clustering, influenced participants’ perception of numerosity. In summary, accumulating evidence suggests that factors other than number alone play a significant role in numerosity estimation tasks, particularly spatial features. As explained above, these findings are in conflict with the prevalent direct ANS theory account, since nonnumerical information should have been removed in the process of creating a numerosity percept. Note that the lack of removing nonnumerical information also questions the claim that number representations are abstract (Brannon & Merritt, 2011), and that an abstract “pure amodal” numerosity representation by the ANS exists. In addition, the amodality assumption is empirically not deeply founded or even contradicted: Only very few studies have investigated numerosity perception in modalities other than the visual or in cross-modal setups (e.g., via tone sequences; Izard et al., 2009), tactile or vibro-tactile stimuli (Tokita & Ishiguchi, 2016; Uluç et al., 2020). Therefore, little is known about similarities or differences of numerosity percepts that are derived from different modalities. More recently, Ziegler and Drewing (2022) used a paradigm of paired presentation of nonsymbolic number stimuli in the haptic and the visual modality comparing participants’ performance in the two modalities. They did not find any associations of the individual participant’s performance between both intramodal numerosity comparison tasks, which they interpreted as a contradictory result to the amodality assumption. Within the modalities, they found effects of spatial configuration, such as the sparsity of a dot array, affecting participants’ numerosity estimate, which additionally questions the abstractness of numerosity representations (Ziegler & Drewing, 2022).

The few cross-modal studies tend to provide counterarguments against abstract amodal representations. Barth et al. (2003) explicitly conducted cross-modal experiments to determine whether the numerosity representation is perceptual or rather abstract. Their assumption was that if a truly abstract representation of (numerical) magnitude exists, there might be little to no cost for cross-modal comparisons relative to unimodal (within-modality, e.g., visual–visual) comparisons in a same–different task. They focused on visual and auditory modalities and different task formats (cf. Dietrich et al., 2015): Participants were exposed to temporally (sequential; visual: flashes, auditory: beeps) or spatially (paired; visual: arrays) presented numerosity stimuli. They determined for each participant which of the two within-modality comparison tasks was more difficult for them (called the “worse unimodal condition”) and compared participants’ discrimination performance in this condition to their performance in the cross-modal condition. Barth et al. (2003) reported that participants’ performance did not significantly differ between the worse unimodal condition and the cross-modal conditions as long as the task format was kept constant. However, participants did perform worse when both modality and task format were crossed (cross-modal and cross-task conditions), which does not seem plausible if true amodal number representation is achieved by filtering out nonnumerical information (cf. Gebuis et al., 2016). Differences in performance between modalities, especially when crossed with presentation format, appear to be a reoccurring pattern across literature (e.g., Anobile et al., 2018). We think, results like those do not fit the theory of an amodal ANS in its “strong” interpretation (i.e., shaping a numerosity percept solely based on number). Tokita et al. (2013) carried out a study investigating the numerosity percepts of individuals with an emphasis on matched task format. They highly standardized a visual and an auditory approximate numerosity comparison task in a sequential paradigm. Participants compared dots or tone sequences in within-modality conditions as well as in a cross-modality condition, all under the same sequential task paradigm. Tokita et al. (2013) found substantial differences in the variability of participants’ performance in the visual and auditory modality. Following these results, they argued against the assumption of a modality-independent numerical representation system.

The above evidence against abstract amodal representations from cross-modal studies relies mainly on sequential presentation of numerosity neglecting influences of spatial information. In this work, we investigate cross-modal approximate numerosity perception using spatial dot arrays in the haptic and the visual modality. Spatial (topological) factors (e.g., sparsity, field area, convex hull, density of dot arrays; cf. Clayton et al., 2015; DeWind et al., 2015) have not been extensively evaluated in cross-modal studies. However, spatial factors have been repeatedly found to be a significant factor that affects participants’ performance within unimodal tasks (Clayton et al., 2015; DeWind et al., 2015; Gebuis & Reynvoet, 2011; Ziegler & Drewing, 2022). In comparison to surface-area-related factors, that sometimes have been found to be influential (especially in children; cf. Anobile et al., 2018; Tomlinson et al., 2020), spatial factors demonstrate their impact even more consistently. Studies in the visual domain repeatedly have demonstrated that particularly the convex hull of a dot pattern as a spatial factor seems to be informative and a highly relevant contributor for a numerosity estimate (Clayton et al., 2015; DeWind et al., 2015). At the same time, spatial factors such as the convex hull can be well presented and perceived both to the haptic and the visual modality and hence could provide a potent cross-modal influence on numerosity.

The haptic modality allows the construction of stimuli that preserve the spatial information in the task, e.g., in form of massed arrays of stimuli similar to visual arrays. In a cross-modal number matching task, we tested whether spatial information from one modality (haptic) is transferred into another modality (visual) along with the information about numerosity and used in a numerosity task. With conducting a cross-modal study and focusing explicitly on spatial information influences, we try to take the amodality assumption of the ANS to a strong test. We argue that if the classical ANS assumptions would apply—that is, numerosity is (directly) processed by the ANS and the amodal numerosity percept arises due to a removal of nonnumerical cues, spatial information would not affect the performance of participants when comparing numerosity across modalities.

We designed a cross-modal number matching task, in which participants perceive a numerosity haptically via an array of dowels and then ask them to match the extracted numerosity to one of two visually presented dot arrays. Here, we vary if the matching (correct) visually presented dot array is spatially identical to the haptic pattern or a random arrangement of the correct number of dots varying in spatial attributes of the stimulus pattern. The distractor dot array is varied in number, resulting in different ratios, which determine the difficulty of the trial. This allows us to address the following questions:

- Are individuals able to use *numerical* information extracted from haptically presented source material and match this information to visually presented target stimuli (cross-modal transfer)?

and

b) is *spatial* information extracted along with number, used in the cross-modal numerosity matching task, and thus affects the responses in the cross-modal numerosity matching task?

Addressing the first question is a confirmation that cross-modal magnitude comparison is possible (as in, e.g., Anobile et al., 2018; Barth et al., 2003; Gallace et al., 2007), but with the advancement that rarely investigated modalities are tested in a cross-modal setup with simultaneously presented source numerosity (dot arrays). This provides the necessary fundament for the second question, that additional information besides numerosity is extracted and used by participants. This would contrast the direct ANS model and widens perspective for alternative proposals.

## Methods

### Participants

We used G*Power (Version 3.1; Faul et al., 2009) to estimate the required sample size of 44 participants to achieve a power of .95 for a medium sized effect (*f* = .25) in a 2 × 5 repeated-measures analysis of variance (ANOVA). We recruited 50 undergraduates (37 females, mean age *M* = 23.22 years, *SD* = 4.48). All participants were healthy and without any impairments or injuries that influenced their touch sensitivity. Participants had normal or corrected-to-normal vision. Forty-three participants were right-handed, and seven were left-handed.

Every participant gave informed written consent to the study prior to the experiment. Consent followed the Declaration of Helsinki (World Medical Association, 2013) and was approved by the local ethics committee of Fachbereich 06 of the Justus-Liebig-University Gießen (LEK-FB 06). Due to the COVID-19 pandemic, protective measures for participants and the experimenter were implemented that complied with the local university guidelines. None of the protective measures compromised the experimental implementation.

### Cross-modal number matching task

In the cross-modal number-matching task, participants compared a haptically presented numerosity (dowel array) to two visually presented numerosities (dot arrays). The participant was instructed to decide which of the two visually presented numerosities contained the same number of dots as in the (immediately) prior presented haptic dowel array. We varied whether the visual target stimulus either is a random arrangement of dots (spatially random [SR]) or matches the exact spatial pattern of the haptic stimulus (i.e., being spatially identical [SI]). Furthermore, we varied the numerosity ratio between the target stimulus and the distractor stimulus so that five different levels of difficulty (1.11, 1.14, 1.20, 1.33. 2.00) were implemented.

While the visual target stimulus in the SI condition takes on its spatial features from the haptic stimulus, we utilized the degrees of freedom in stimulus placement in the SR condition to additionally manipulate spatial attributes of the pattern to generate spatially congruent and incongruent trials. As a key metric for spatial influence, we used a compound index, ”spacing,” defined by DeWind et al. (2015) as the product of convex hull and sparsity of a stimulus array. The convex hull can be illustrated as a polygon defined by a subset of elements (dots) containing all elements of the set. Sparsity is defined by the area of convex hull divided by the number of elements in the stimulus array (DeWind & Brannon, 2016). The spatial metric of the haptic stimulus array can be either congruent to the visual target stimulus or to the distractor stimulus (see Experimental Procedure and Data Analysis sections). In a congruent trial, the difference in the variable “spacing” of the haptic stimulus pattern and the target visual stimulus pattern is smaller than the difference in “spacing” of the haptic stimulus pattern and the distractor stimulus pattern. A trial is incongruent if there is a smaller difference in the space metric (= “spacing” compound index) between the distractor visual stimulus pattern and the haptic stimulus pattern than between target visual stimulus and haptic pattern. With this definition, the spatial influence of the pattern can be seen as a facilitating or conflicting factor to the correct response.

#### Experimental setup

**Fig. 1:**
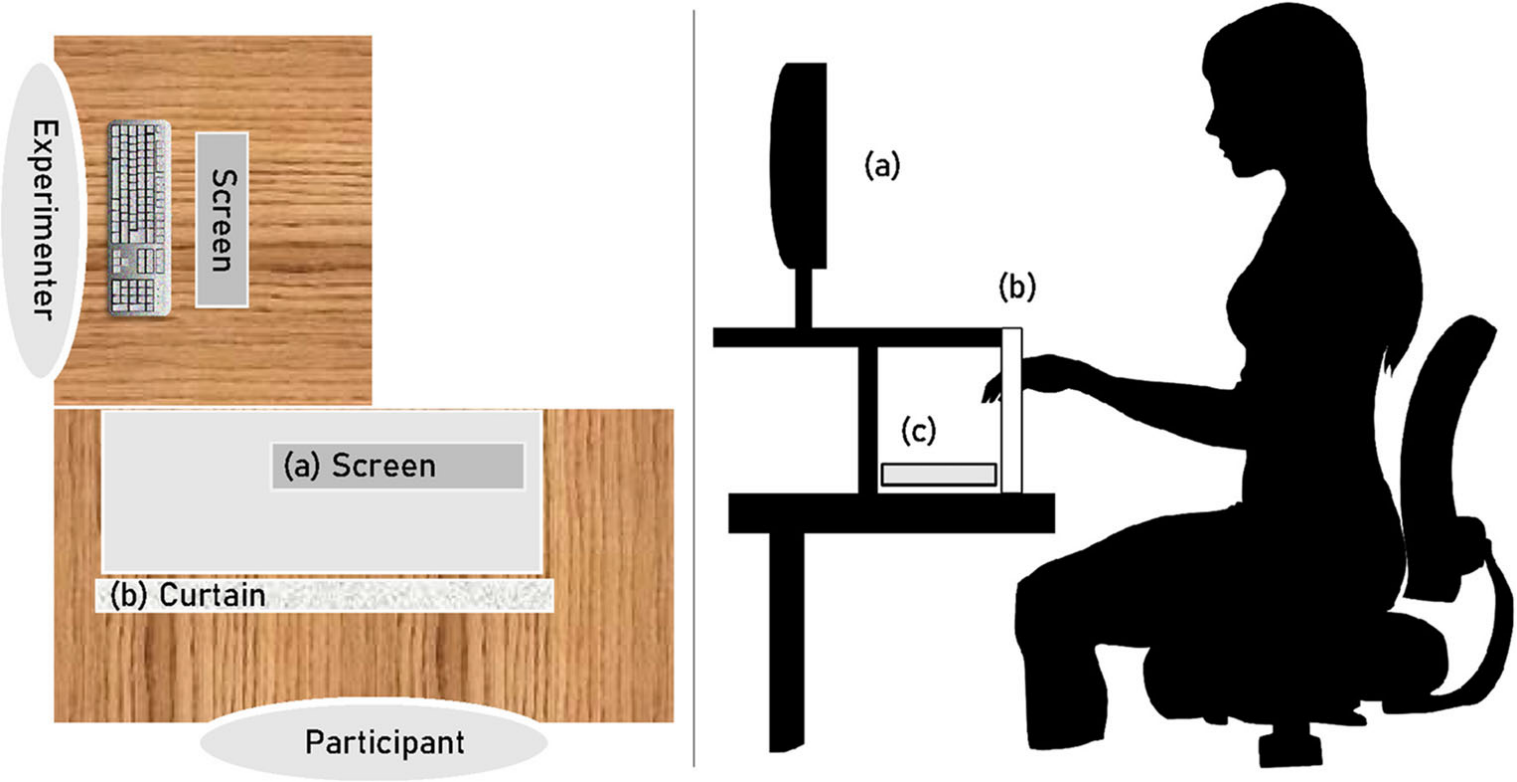
Illustration of the experimental setup. *Note.* Left image shows a top-down view of the setup. Right image shows a cross-section view of the setup and how the participants engaged in the task. **a** Participant’s screen for visual stimuli; **b** curtain that prevents the participant from visually inspecting the haptic stimulus; **c** haptic stimulus

The experiment took place in a quiet, darkened room in the Faculty of Psychology at Justus-Liebig-University Gießen. Participants sat in front of a 22-inch monitor (visible screen: 47.4 cm × 29.6 cm, brightness: 250 cd/m^2^, resolution: 1,680 × 1,050, refresh rate: 60 Hz) at 60-cm viewing distance in front of a table. The monitor was placed on a height-adjustable construction that was put on top of the table (see Fig. 1), allowing participants to see the visually presented stimuli at eye level and giving enough space below to comfortably explore the haptic stimuli. We adjusted the screen height individually, so that participants looked directly into the center of the screen with upright head posture. The experimenter sat to the left of the participant and could monitor and track the hand during the participant’s haptic exploration process. An adjustable opaque piece of fabric (120 × 50 cm) prevented the participant from seeing the haptic stimuli during the experimental trials. Participants could rest their hands under the construction during the experiment. The haptic stimuli were presented 6 cm to the left or 6 cm to the right of the body midline, depending on the participants preferred handedness. The stimulus was locked in place during the trial by a fixture mounted to the table.

#### Haptic stimuli

**Fig. 2:**
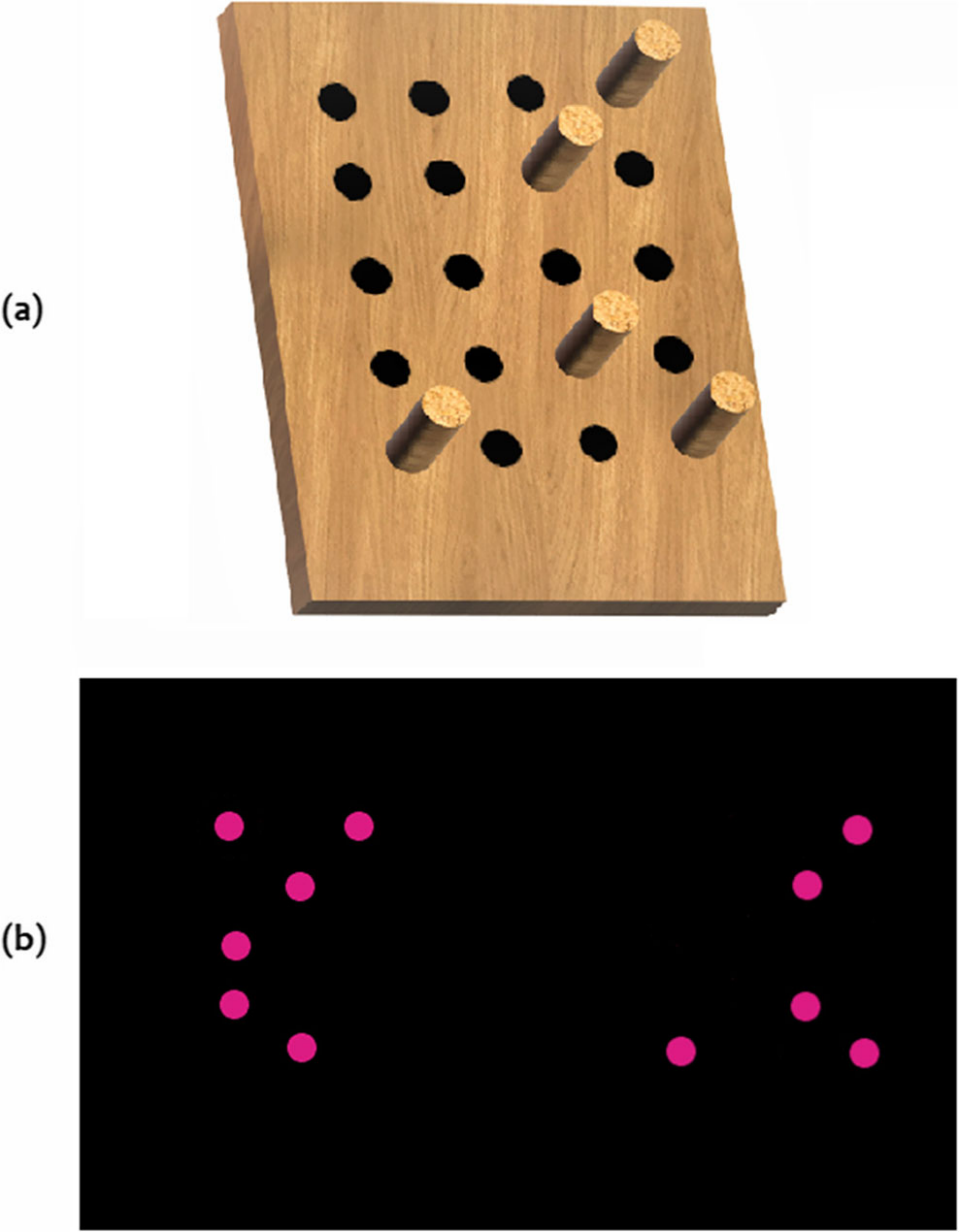
Examples of haptic and visual stimuli used in the experiment. Note. (***a***) Haptic stimulus. (***b***) Visual stimulus. Haptic pattern matches the visual stimulus on the right side, which displays the identical pattern of dots (SI condition). (Color figure online)

The haptic stimuli were wooden panels (length: 119 mm, width: 99 mm, height: 21 mm) made from sanded down veneer plywood (“multiplex”), which could be equipped with varying numbers of wooden dowels. The dowels functioned as single enumerable entity. We prepared 20 slots (diameter: 6 mm, depth: 10 mm) for a panel, which were organized in a 5 × 4 rectangular scheme. The center-to-center distances between the slots were 10 mm, resulting in a 70 mm × 55 mm rectangular area (see Fig. 2). The distance of 10 mm was chosen to enable distinct discrimination of individual dowels in the palm of the hand (Craig & Lyle, 2001). As a reference for the size of the panel and the distances of the slots, we used a glove size S (17.5 cm of hand circumference) to ensure that also individuals with small hand sizes could quickly palpate the stimulus. Any slot could contain an industrial wooden dowel (diameter: 6 mm, length: 30 mm). Each dowel thus protruded 20 mm from the panel. The haptic stimulus could be prepared rapidly during the experiment for the subsequent trial, due to a custom-written software that assisted the experimenter by visualizing the current and next trial. The panels were clipped to the table to ensure stable hold during the exploration phase. In each trial, a panel contained a dowel (dot) array ranging from 5 up to 10 entities that were randomly (pseudorandomly in the “spatially randomized” condition) selected by the custom-written C++ program. Any position had an equal probability of being selected for the current trial. If a trial did not meet the criteria for the “spatially identical” (SI) condition (i.e., the random pattern was not congruent or incongruent in the spatial attributes), the program recalculated the trial until it matched the criteria (exhaustive search method).

#### Visual stimuli

Visual stimuli were two simultaneously presented dot arrays on each side of the screen. The arrays were presented 105 mm apart from each other and were clearly distinguishable as separate arrays. A single dot had a diameter of 6 mm on the screen. The minimum center-to-center distance between the dots corresponded with the haptic stimuli and was at least 10 mm on the display. Each array displayed 5 up to 10 magenta-colored dots on black background (see Fig. 2). Possible positions of the dots were arranged in direct correspondence to the possible position of dowels in the wooden panels (5 rows × 4 columns).

### Experimental procedure

**Fig. 3:**
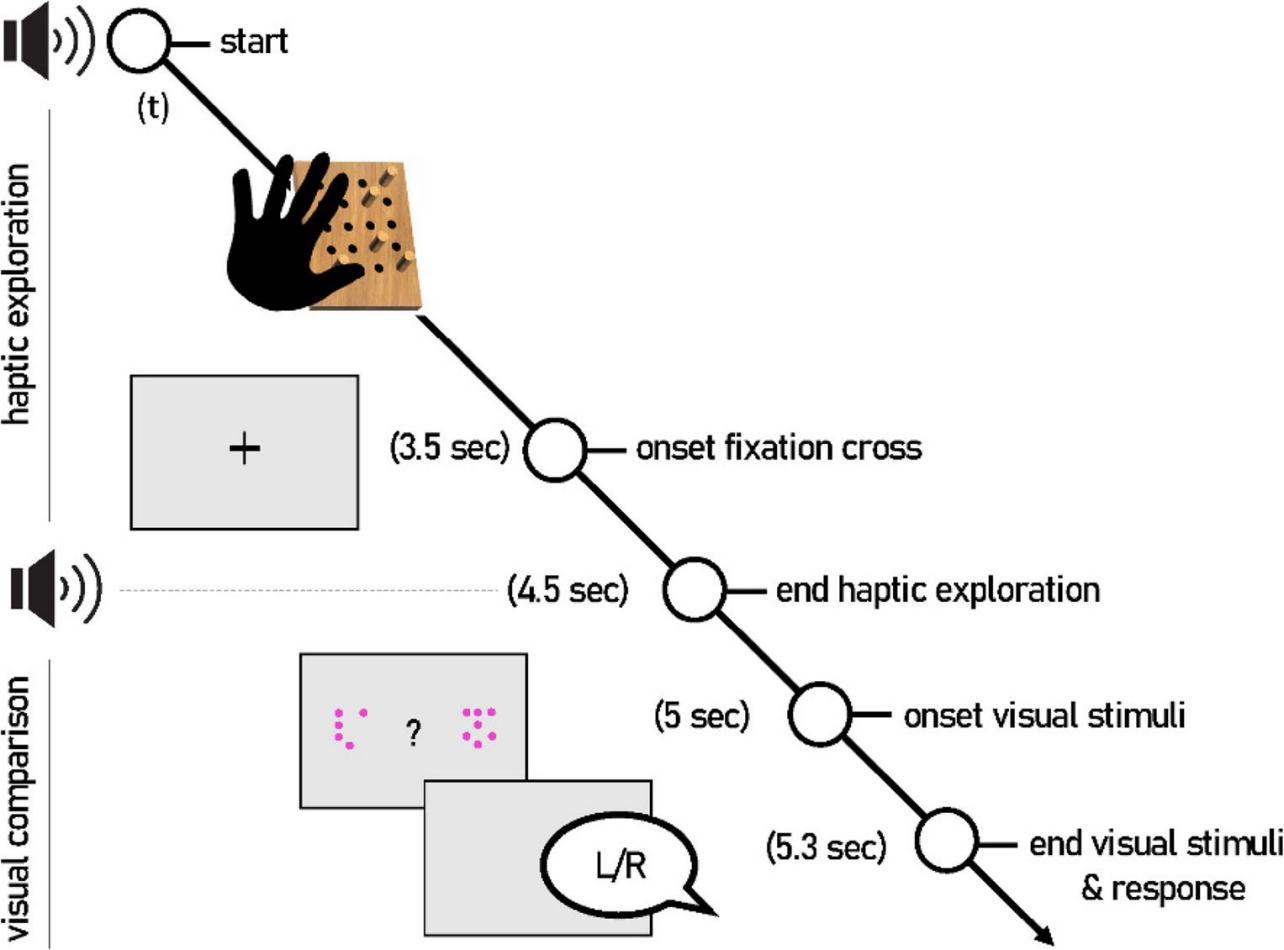
Illustration of the procedure in the cross-modal matching task

Each trial started with the participant placing the preferred hand onto the haptic stimulus through the curtain underneath the construction. The experimenter started the trial when the participant touched the stimulus material. A beep tone (duration: 500 ms, pitch: 523 Hz) signaled the participant to start the exploration phase. The participant explored the dowels with the palm of the dominant hand for a total of 4.5 seconds. Participants were prompted to place their hand onto the stimulus several times within the time limit to ensure that they touched all dowels with the palm of their hands, which was monitored by the experimenter. After 3.5 s during the haptic exploration phase, a white fixation cross appeared on the screen. At 4.5 s, a second beep (duration: 100 ms, pitch: 523 Hz) signaled the participant to lift the hand from the haptic stimulus (transition phase). 500 ms later, the fixation cross vanished and two dot arrays were presented for a total of 300 ms, sufficient to give an approximate estimate of the numerical quantity presented. Participants were instructed to choose the side that matched the number of dowels as felt on the panel by verbal announcement “left” or “right,” respectively. The response was documented by the experimenter and the next trial began. The full procedure is illustrated in Fig 3.

The experimenter prepared (i.e., equipped the panel with dowels) and exchanged the haptic stimulus in each trial according to a custom-written C++ script. The haptic dowel array was presented to the experimenter by a second screen, which was not visible to the participant. A total of 80 trials were presented. The trial list was shuffled randomly for each participant. Participants were instructed that there is always one visual dot array with the exact same number of dots as dowels and that the other one contained either fewer or more dots.

The brief presentation time of the stimuli (signal to uncover at 4.5 seconds haptic; 300 ms visual) served to prevent the participant from counting the dowels/dots. Pretests were used to determine the appropriate presentation times. Participants were instructed explicitly to base their decision on number. Some participants asked whether the arrangement of the dowels and the arrangement of dots in the dot array might play a role. If this question was posed, the experimenter gave the following answer: “The correct answer is the dot array with the same amount of dots as dowels felt previously. I am not allowed to give any further information about the stimuli.” Participants were not given any feedback on whether their answer was correct or not.

In half of the trials, the visual target dot array of correct numerosity was identical (i.e., in numerosity and spatial arrangement) to the dowel array of the haptic stimulus (“spatially identical” [SI]). In the other half of the trials, the correct answer matched only the number of the dowel array, but not the spatial pattern (“spatially randomized” [SR]), which was a spatially random arrangement of dots. Still, the stimuli generating formula allowed balancing the convex hull of the visual stimuli in the “spatially randomized” condition, so that in half of these trials the matching stimulus had a convex hull larger than the distractor stimulus and in the other half a convex hull smaller than the distractor stimulus.

The distractor stimulus in the visual modality was varied in different ratios relative to the haptic stimulus. We applied five different comparison ratios within the experiment, 10:9 (1.11), 8:7 (1.14), 6:5 (1.20), 8:6 (1.33), and 10:5 (2.00). These ratios determine the difficulty if the comparison process is solely based on number. There are easy to distinguish alternatives, for example, haptic stimulus contains 5 dowels, Visual Stimulus 1 shows 5 dots (target), Visual Stimulus 2 shows 10 dots (distractor), and difficult to distinguish alternatives (haptic stimulus contains 9 dowels, Visual Stimulus 1 shows 9 dots (target), Visual Stimulus 2 shows 10 dots (distractor). Each ratio was tested 16 times (8 times in each pattern condition). In 50 % of the trials, the target visual stimulus displayed a lower number of dots than the visual distractor stimulus, and in the other 50 % of trials a higher number of dots. The side on which the “correct” dot array was presented was balanced across trials as well. Two pattern conditions (spatially identical, spatially randomized), 5 ratio conditions, 2 possibilities of whether choosing the higher or lower number as correct target stimulus, 2 convex hull conditions (smaller/larger), and 2 possible sides for the correct answer, resulted in 80 possible combinations (2 × 5 × 2 × 2 × 2 = 80), all of which were tested in a randomized order. The experiment took about 45 minutes to complete.

### Data analysis

For each participant, we calculated the mean of correct answers for each ratio and the two levels of three variables—that is, pattern (SI, SR), magnitude (larger number, smaller number), and bias towards a side (left, right). The factor “magnitude” (larger number, smaller number) allows to check for potential effects on answers in dependence on the relative target magnitude (i.e., whether the target stimulus has the smaller or larger number than the distractor). The bias analysis served as a control condition to detect whether participants had unforeseen tendencies towards a side, regardless of which dot pattern was shown (left side, right side).

We applied arcsine transformation (Cohen et al., 2014) for the aggregated correct answers as preparation for a repeated-measures ANOVA (rmANOVA) with the within-subject variables ratio (2.00, 1.33, 1.20, 1.14, and 1.11) and pattern (spatially identical, spatially randomized). Two additional rmANOVAs with the within-subject variables “ratio” (2.00, 1.33, 1.20, 1.14, and 1.11) and the factors “magnitude” (larger, smaller number) and “bias side” were conducted, respectively. For each rmANOVA, we applied Greenhouse–Geisser correction (Geisser & Greenhouse, 1958) in case of any sphericity violations.

To investigate spatial influence, represented by the product of sparsity and convex hull of a stimulus pattern, on participants’ answers, we analyzed the trials of the “spatially randomized” pattern condition with a generalized linear model (probit link function with iteratively reweighted least squares). The correct answers served as response variable and the variables “ratio,” “spatial congruency” (incongruent/congruent), “magnitude,” and the bias variable (“bias side”) as well as a variable accounting for interindividual differences in performance (“individuals”) as predictors. We use the compound of sparsity and convex hull as an index for spatial influence as it is a relative measure that includes the key spatial features of a stimulus array (DeWind et al., 2015). A trial is spatially congruent if the following condition is met:

(*Haptic*_*space*_ − *VisualTarget*_*space*_) < (*Haptic*_*space*_ − *VisualDistractor*_*space*_) (1)Spatial congruency is therefore a dichotomous variable (congruent, incongruent) reflecting congruency of spatial attributes between the haptic pattern and the visual target pattern.

The data preparation, computation, and visualization of the data were performed using R (Version 4.1.0; R Core Team, 2019).

## Results

Table 1 shows the relative frequency of correct trials from all 50 participants sorted by ratio and pattern condition (spatially identical/spatially randomized).

**Table 1:** Relative frequencies of correct trials per ratio and pattern in the complete sample

Ratio	Pattern	*N*	*M*	*SE*
1.11	SI	50	.695	.030
1.11	SR	50	.522	.025
1.14	SI	50	.718	.022
1.14	SR	50	.542	.025
1.20	SI	50	.725	.026
1.20	SR	50	.570	.025
1.33	SI	50	.748	.025
1.33	SR	50	.672	.025
2.00	SI	50	.922	.012
2.00	SR	50	.877	.018

*Note*. “SI” pattern (spatially identical) refers to the condition when the haptic dowel array and the correct visual dot array match in numerosity and also in spatial arrangement. “SR” (spatially randomized) pattern refers to the condition when the haptic dowel array and the correct visual dot array solely match in numerosity but not in spatial arrangement

**Fig. 4:**
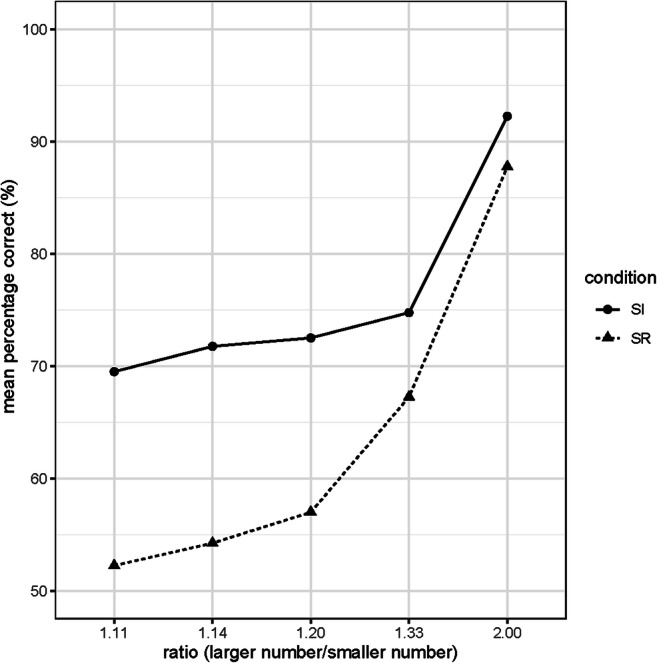
Mean percentage correct per ratio and pattern in the complete sample. *Note*. “SI” refers to the spatially identical pattern condition when the haptic dowel array and the correct visual dot array match in numerosity and also in spatial arrangement. “SR” refers to the spatially randomized pattern condition, when the haptic dowel array and the correct visual dot array solely match in numerosity but not in spatial arrangement. *N* = 50

Figure 4 shows the mean percent correct responses of the variable pattern across all applied ratios (2.00, 1.30, 1.20, 1.14, and 1.11).

### Ratio and pattern

The main results of the repeated-measures ANOVA revealed a significant main effect for the factor ratio (2.00, 1.30, 1.20, 1.14, and 1.11) with *F*(4, 196)= 59.246, *p* < .001, ${\upeta}_p^2$ = .547 as well as a significant main effect for the factor pattern (spatially identical, spatially randomized) with *F*(1, 49) = 59.571, *p* < .001, ${\upeta}_p^2$ = .549. Participants showed a higher accuracy rate with higher ratios. The accuracy rate is higher for spatially identical patterns (see Fig. 4). This trends across all ratios. There is also a significant interaction between ratio and pattern *F*(1, 196)= 2.791, *p* < .001, but with a smaller effect, ${\upeta}_p^2$ = .054.

### Magnitude and bias analysis

We ran repeated-measures ANOVAs to check for effects of number magnitude (larger number, smaller number) and of a potential response bias—that is, a bias towards a side (“bias side,” left/right) among the visual stimuli. The bias analysis indicates there was no tendency in responses towards a side, *F*(1, 49) = 0.014, *p* = .906, ${\upeta}_p^2$ = .000. Furthermore, there is no interaction effect between the factor “bias side” and “ratio” *F*(1, 196) = 1.098, *p* = .359, ${\upeta}_p^2$ = .022. The descriptive statistics are given in Table 2.

**Table 2:** Relative frequencies of correct trials per ratio and magnitude in the complete sample

Ratio	Magnitude	*N*	*M*	*SE*
1.11	LN	50	.610	.026
1.11	SN	50	.608	.028
1.14	LN	50	.605	.024
1.14	SN	50	.655	.025
1.20	LN	50	.635	.028
1.20	SN	50	.660	.024
1.33	LN	50	.665	.028
1.33	SN	50	.755	.024
2.00	LN	50	.880	.019
2.00	SN	50	.920	.015

*Note*. *LN* larger number; *SN* smaller number

The analysis for the factor “magnitude” revealed no significant result, *F*(1, 49) = 3.731, *p* = .059, ${\upeta}_p^2$ = .071; as well as no significant interaction between the factor ”magnitude” and “ratio” *F*(1, 196)= 1.602, *p* = .175, ${\upeta}_p^2$ = .032.

The corresponding descriptive statistics are given in Table 3.

**Table 3:** Relative frequencies of correct trials per ratio and side in the complete sample

Ratio	Side	*N*	*M*	*SE*
1.11	L	50	.583	.026
1.11	R	50	.635	.029
1.14	L	50	.635	.024
1.14	R	50	.625	.022
1.20	L	50	.650	.024
1.20	R	50	.645	.022
1.33	L	50	.715	.023
1.33	R	50	.705	.023
2.00	L	50	.912	.013
2.00	R	50	.887	.016

*Note*. “Side” (Left, Right) refers to the response (i.e., left or right side)

Since the analysis for the variable “magnitude” turned out to be at least marginally significant in the overall sample, *F*(1, 49) = 3.731, *p* = .059, we checked whether this variable is relevant for the subset of trials in the SR condition which is designated for the logistic regression analysis. We found that whether the correct answer was the smaller or the higher number is a significant aspect to consider, *F*(1, 49) = 5.448, *p* = .024. We therefore ran two separate models for the logistic regression, one for trials, in which the higher numerosity was the correct answer and a complementary one, in which the smaller numerosity was the correct answer.

### Logistic regression analyses

Within the subset of trials of the “spatially randomized” pattern condition, we distinguished trials where the haptic stimulus was spatial congruent versus spatial incongruent to the visual target stimulus (relative to the relation between haptic stimulus and visual distractor stimulus).

The results of this analysis is given in Table 4. We ran separate models for trials in which the correct target was the higher number (Model A) and trials in which the correct target was the smaller number (Model B). We added the predictors ratio (2.00, 1.30, 1.20, 1.14, and 1.11), spatial congruency (congruent, incongruent) as well as the ID variable (“individuals”) representing interindividual differences in participants.

**Table 4:** Model coefficients (Models A and B) and statistics for the logistic regression of correct answers on ratio and spatial congruency

Predictors	β	*Z*	*SE*	*p*
Model A				
Ratio	1.107	7.932	0.139	<.001***
Spatial congruency	0.247	2.997	0.083	<.010**
Individuals	−0.003	−0.876	0.003	.381
Intercept	−1.256	−6.102	0.206	<.001***
Model B				
Ratio	1.455	8.905	0.163	<.001***
Spatial congruency	−0.111	−1.307	0.085	.191
Individuals	0.000	−0.065	0.003	.948
Intercept	−1.419	−6.185	0.229	<.001***

*Note.* Model A refers to all trials in the “spatially randomized” pattern condition in which the correct target was the higher number; Model B refers to all trials in this condition in which the correct target was the smaller number. “Ratio” includes all ratios 2.00, 1.33, 1.20, 1.14, and 1.11. “Spatial congruency” is a dichotomous variable reflecting congruency in spatial features between the haptic pattern and the visual target numerosity; the predictor “individuals” accounts for interindividual differences in the participants’ task performance

**p ≤ .01. ***p ≤ .001

#### Model A

The AIC (Akaike information criterion) of the regression Model A is 1,269.4 with a null deviance of 1,339.3. The estimated pseudo-*R*-squared of the model is at 0.102 (Cragg–Uhler), which is acceptable but gave us reason to ensure the validity of the model fit with the method proposed by Hosmer and Lemesbow (1980). The Hosmer and Lemesbow test does not indicate a poor model fit, χ = 6.930, *df* = 8, *p* =.544. The logistic regression model shows that the number ratios affect the decision for a correct answer the most. Higher ratios (e.g., 2.0) are more likely to provoke a correct answer. Additionally, if a trial showed spatial congruency it facilitated a correct response (see Table 4). The interindividual difference in performance did not show a significant impact within the logistic regression model.

#### Model B

The AIC (Akaike information criterion) of the regression model B is 1,181.6 with a null deviance of 1,274.0. The estimated pseudo-*R*-squared of the model is at 0.133 (Cragg–Uhler), which is acceptable as well. The Hosmer and Lemesbow test does not indicate a poor model fit, χ = 9.124, *df* = 8, *p* =.332. The logistic regression model shows that the number ratios affect the decision for a correct answer the most. Higher ratios (e.g., 2.0) are more likely to provoke a correct answer. Spatial congruency did not facilitate a correct response (see Table 4) and was not a significant predictor in Model B. The interindividual difference in performance did not show a significant impact within the logistic regression.

## Discussion

In the present study, we examined the ability to compare haptically and visually perceived numerosity from dot arrays in a cross-modal number matching task. We focused on possible effects of spatial information provided by the dot/dowel patterns within this paradigm. We therefore had participants decide which of two visually presented numerosities matched the previously haptically perceived numerosity; we varied whether the target visual stimulus was spatially identical (SI) to the haptic stimulus or whether it displayed a random arrangement (SR) of the same number of dots. The visual distractor stimulus was a numerosity that determined the difficulty of the comparison. The number ratio difficulty ranged from 2.00 (easy) to 1.11 (hard). We furthermore varied whether the visual target stimuli with a spatially randomized pattern (SR) displayed a pattern that is congruent or incongruent to the spatial features of the haptic pattern. We expected that if numerical information is the only processed information in this task, which is implied by the “strong” assumption of the ANS theory, spatial information would not affect the accuracy rates under the variation of the dot patterns (SI/SR). However, our analyses showed a remarkable and significant main effect of the factor “pattern” towards a processing advantage for spatially identical patterns, implying that spatial information is extracted and maintained throughout cognitive processing and has a significant impact on a participant’s response. Furthermore, our logistic regression model indicates, at least conditionally, that spatial variables also affect response behavior in the SR condition of our cross-modal approximate number matching task. This demonstrates that participants extracted and used spatial information, besides numerosity, from the given stimulus material even across modalities.

A prerequisite for this study was to confirm that individuals are able to use numerical information extracted from haptically presented source material and match this information to visually presented target stimuli. The accuracy rates in both task conditions show ratio dependency, which is indicative for numerosity processing (Feigenson et al., 2004; Spelke & Kinzler, 2007). Therefore, we can assume that participants compared the number of stimuli, just as instructed, which is consistent with results of previous cross-modal studies (Barth et al., 2003; Gallace et al., 2007; Tokita et al., 2013). What stands out in this study is that in addition to the numerosity of the haptic stimulus, spatial information of the stimulus arrangement was apparently extracted along with number. This result extends previous work by the aspect that spatial information can influence a person’s numerosity percept in a cross-modal setup, which has already been shown in intra-modal numerosity tasks (Clayton et al., 2015; DeWind et al., 2015; Ziegler & Drewing, 2022). In both conditions, the spatially identical and spatially random pattern, spatial information affected the responses in the cross-modal numerosity matching task. A match in numerosity between the haptic and the visual modality with an identical stimuli arrangement showed the strongest effect and facilitated participants’ accuracy significantly. We furthermore demonstrated that correct numerosity discrimination is more likely when the haptic stimulus is congruent in spatial attributes to the visual target stimulus, when the target numerosity was the larger number. The proportions of the effect of ratio and spatial congruency onto the discrimination performance are similar to the effects of numerosity and space found in the haptic-visual intramodal numerosity comparison task by Ziegler and Drewing (2022), which indicates that the same factors contribute to the numerosity percept. Furthermore, the finding of spatial congruency is in accordance with studies that demonstrated intramodal spatial congruency effects (i.e., there is a facilitating effect when spatial and numerical attributes match; Gilmore et al., 2016). Contrary to our expectations, the congruency effect did not show in trials that had the smaller numerosity as target, so the interpretable effect is restricted to the subset of trials in which the target numerosity was the larger one. We assumed that a congruency effect would likewise occur when a smaller “space” goes along with “smaller” numerosity—an assumption we cannot verify in our data.

However, both of these findings, the spatial effect in the SI and the SR condition, are contrary to the assumption that numerosity is achieved by a process that filters out nonnumerical information, which has been proposed by some direct models of approximate numerosity processing (Brannon & Merritt, 2011; Dehaene & Changeux, 1993). Therefore, explanations of the traditional direct ANS theory in how numerosity becomes an amodal representation are challenged as well. The idea of a cognitive system that filters out nonnumerical information to create a pure numerosity percept seems not plausible with our and previous findings.

Nevertheless, it is reasonable to assume that a common representational basis of stimulus information exists in order to compare the magnitudes extracted from different modalities. Our results encourage speculation about the unknown processes and factors involved in numerosity perception and how the results might be embedded in alternative theories. We think key aspects to consider in this discussion and in future work is the extent to which spatial information interacts with numerosity information, whether an integrated numerosity percept is constructed or whether number and spatial information are maintained independently.

Barth et al. (2003) argued for an abstract common magnitude representation that allows comparison between modalities. We partially agree on this, but with the caveat that the numerosity percept is either influenced by or even dependent on spatial factors, or that spatial factors are extracted alongside number and used in the decision stage. The interaction effect between the factor ratio and the pattern condition in our study furthermore implies that the difficulty of a trial, which should be exclusively determined by the ratio, is further modified by the spatial information given. The ratio dependency points towards a mechanism in which spatial information directly shapes the final numerosity percept, which would be in accordance with indirect models of numerosity estimation (cf. Allïk & Tuulmets, 1991). However, the salient effect of spatial information and consistently better performance across ratios in the SI condition also allows for the plausible interpretation that the numerosity percept and the spatial percept are kept independently but within a shared metric, and that each factor contributes to the decision of choosing the matching “numerosity.” In this perspective, the decision whether a numerosity is the same is influenced by these two (and additional) factors, without an irreversible merge into a single numerosity percept. This would follow the interpretation by Lourenco and Aulet (2022) that a shared but distinct representation throughout cognitive processing exists. What remains unclear is to what extent the numerosity factor might be dependent on spatial information assuming that numerosity is derived from topological information, or whether number is a primary feature (cf. Aulet & Lourenco, 2021; Gebuis et al., 2016; Lourenco & Aulet, 2022). In principle, there might be the possibility of an integrated numerosity percept composed of number, space, and presumably area factors, as well as the alternative possibility that space and numerosity are extracted and maintained as distinct multidimensional percepts that can be read out voluntarily, as suggested by Lourenco and Aulet (2022).

Marinova et al. (2021) recently suggested that there might be more than just one way in how numerosity is estimated and that humans are capable of using direct and indirect estimation mechanisms depending on the individual and context. In our context, it would be reasonable to assume that different stimulus material also evokes different information processing within the participant. For example, in our SI condition, participants might have been provoked to emphasize on the spatial information, whereas in the SR condition, due to the lesser spatial salience of the stimulus material, decision making relied more extensively on the numerosity factor of the percept. Still, spatial information remains relevant in the SR condition, but only insofar as the mechanism that derives numerosity from the stimulus pattern is affected by the spatial properties. Additionally, the different effects of spatial congruency hint towards different processing mechanisms within this condition. Ernst and Banks (2002) describe that information integration from different modalities is weighted by its reliability. Cues that are more reliable can “capture” a percept (or decision process). Transferring to our results, spatial features and number features within a task would compete for weighting within the final percept (or at least for the terminal decision, if one assumes that there is no final integration). We think this idea would be plausible and in consonance with the data we present here.

The idea that multiple mechanisms for approximate numerosity estimation exist assumes that there is domain-specific processing of the stimulus material in participants. A limitation of our study might be that participants could have used strategies that bypassed “numerosity” processing and based their decision on other factors, such as a “pattern match” or “shape recognition” of dot arrays. In this view, the results would not directly compromise the traditional direct ANS assumptions, as participants would have just used a strategy to match the task affordances, but cognitive processing could still appear to be rather domain general than domain specific. It is well known that participants supplement their decisions in numerosity comparison tasks with different strategies (Dietrich et al., 2019; Roquet & Lemaire, 2019). A strategy that Roquet and Lemaire (2019) named a “shape”-based strategy could question the striking effect we found for the pattern condition. In this strategy, participants respond to a recognizable shape rather than to specific features like numerosity or space (even though topographic information is still used). Although we cannot completely rule out the possibility that participants occasionally used this strategy, it is very unlikely to explain the effect. Shape recognition was reported as least frequent (0.6 %) in Roquet and Lemaire (2019) in conditions that allowed this strategy to be used even better (intramodal numerosity comparison) than in our setup. However, participants may have selectively used more of what Dietrich et al. (2019) classified as a “numerosity-based strategy” in the “randomized pattern” condition and leaned more toward a “visual strategy” in the “spatially identical pattern” condition. Roquet and Lemaire (2019) used similar terms of “numerical” and “visual” strategies for classification. In our case, visual strategies should be referred to as “topographic” strategies—because the source modality is perceived haptically.

Besides the domain specific processing, other more general aspects in human processing are also involved in a participant’s performance, which also might partially explain the performance difference in the SI and SR conditions—for example, the availability of attentional resources. A convincing body of evidence indicates that attentional resources are (at least) partially shared across the visual and haptic modality for tasks that require spatial attention or object-based attention (Wahn & König, 2017). A cross-modal facilitation of performance from one modality to the other due to spatial information could partly be an effect of the shared spatial attention resources between the two modalities (visual, haptic): When participants compared the stimuli (i.e., comparing the haptic percept with the two visual stimuli), a shared spatial pattern of attentional foci between the modalities might have enhanced areas in the spatially identical condition in vision that are already represented from the extraction of the haptic stimulus. That is, visual focal attention in the spatially identical condition might be better tuned to capture the visual stimulus than in the spatially random condition, and thus might have contributed to a better performance in the former condition. Put in other words, in the spatially random condition, the visual and haptic stimuli share less spatial structure in attentional foci as compared to the spatially identical condition and therefore might not enhance performance to the same extent. Our number matching task could have involved both, object-based attention, in which number is the object (analogous to a target letter or a color; cf. Alvarez, 2011), as well as spatial attention, which links to the spatial information of the dot patterns. Both types of attention have recently been demonstrated as influential in visual numerosity estimation tasks (Pomè et al., 2021). Therefore, both could have contributed to the decision process within the cross-modal task, especially in our combination of modalities (visual–haptic). The control of attentional processes could be systematically introduced as factor in experiments with approximate numerosity to better disentangle domain general and domain specific factors that contribute to a participants performance. The role of different types of attention and their effect on performance in numerosity estimation has only recently started to become a greater topic and requires further research (Pomè et al., 2021).

Overall, given the ratio-dependent performance in our task, we have good reason to conclude that participants based their decisions directly or indirectly on numerosity. We furthermore wonder whether strategy use limits any interpretations of the results or whether they are indicative of the distinct processing and if attentional resources partially can explain the performance differences as we have previously speculated.

The (approximate) number of multiple objects is just one feature that can be extracted of a dot pattern nearly instantaneously (Zhang et al., 2019). There is evidence that number is extracted as primary feature (Alvarez, 2011; Feigenson et al., 2004). Additionally, as demonstrated in the visual modality, summary statistics (e.g. averages, variances, orientation, or location of are extracted from objects; Alvarez, 2011). Because selective attention is limited, humans remain being capable to represent multiple aspects of stimuli in these abstracted ways, which are also known as ensemble representations (Alvarez, 2011). These ensemble representations can complement the perception (Alvarez, 2011). Numerosity, per se, seems to be processed distinctly from other statistics in the ensemble representation (Utochkin & Vostrikov, 2017; Yu & Zhao, 2015). However, as mentioned before, a stimulus pattern in our task conveys a variety of information that can be perceived and maintained as ensemble representation throughout cognitive processing. Perceiving sensory information from a haptic stimulus pattern presumably underlies the same principles in the ensemble feature extraction. We wonder, if the spatial information we presented in the SI condition of our task could have been represented and later facilitated the performance of performance just as in the visual modality, through a comparison of the available information in an abstracted summary form (Alvarez, 2011; Alvarez & Oliva, 2009). This would seem plausible to us; however, our experiment did not address this question. Further research will be necessary to clarify the how ensemble statistics extracted from one modality translate into performance of other modalities. We think, that there is an interesting opportunity to find out more about the nature of abstract ensemble representations and if they translate between different modalities and ultimately think all these aspects together in a more general framework of human perception.

In summary, we demonstrate that nonnumerical (i.e., spatial, information is used within our cross-modal number matching task to compare numerosities between the visual and the haptic modalities). We argue that spatial influence affects a person’s perception of the given nonsymbolic number stimulus material in our cross-modal matching task, assuming that the final (numerosity) percept includes a composite of number and space (DeWind et al., 2015). We furthermore argue that the so-called approximate number system might be involved in this process, but presumably not according to the strong assumption that this system integrates only numerical information, which has also been questioned in alternative theories and so-called indirect models (cf. Gebuis et al., 2016; cf. Leibovich, Katzin, Harel, & Henik, 2017a). Our data suggests that spatial influence does indeed have a strong impact on participants’ decisions, and therefore data interpretation better fits these alternative proposals. We also found that spatial congruency affects the likelihood of accurate numerosity estimations, but it depends on the context: Congruent spatial information facilitated responses when the target number was the larger one, but no congruency effect was observed when the smaller number was paired with smaller spatial magnitude, which should also have been facilitating according to our reasoning. These different congruency effects could be an interesting topic for further research, as congruency effects could help to understand which model of numerosity estimation might be most appropriate (Lourenco & Aulet, 2022).

We articulate concerns about direct ANS conceptions that are narrow in scope and in conflict with recent evidence showing that a numerosity percept is affected by other factors (Clayton et al., 2015; Tomlinson et al., 2020; Ziegler & Drewing, 2022) and therefore, in our opinion, challenge the abstraction claim of the ANS. Albeit speculative, we highlight a range of possibilities for how numerosity processing can work to enable approximate cross-modal number comparison and also allow for flexible adaptation to environmental demands (e.g., via strategy usage). We think that our results also support the suggestion recently proposed by Marinova et al. (2021) that openness to the idea that there may be more than one way in how numerosity is estimated, depending on the individual and context, would better advance this field of research.

## References

[CR1] Allïk J, Tuulmets T (1991). Occupancy model of perceived numerosity. Perception & Psychophysics.

[CR2] Alvarez GA (2011). Representing multiple objects as an ensemble enhances visual cognition. Trends in Cognitive Sciences.

[CR3] Alvarez GA, Oliva A (2009). Spatial ensemble statistics are efficient codes that can be represented with reduced attention. Proceedings of the National Academy of Sciences.

[CR4] Anobile G, Arrighi R, Castaldi E, Grassi E, Pedonese L, Moscoso PAM, Burr DC (2018). Spatial but not temporal numerosity thresholds correlate with formal math skills in children. Developmental Psychology.

[CR5] Aulet LS, Lourenco SF (2021). Perceived number is not abstract. The Behavioral and Brain Sciences.

[CR6] Barth H, Kanwisher N, Spelke E (2003). The construction of large number representations in adults. Cognition.

[CR7] Barth H, La Mont K, Lipton J, Dehaene S, Kanwisher N, Spelke E (2006). Non-symbolic arithmetic in adults and young children. Cognition.

[CR8] Bertamini M, Zito M, Scott-Samuel NE, Hulleman J (2016). Spatial clustering and its effect on perceived clustering, numerosity, and dispersion. Attention, Perception, & Psychophysics.

[CR9] Bisazza A, Gatto E (2021). Continuous versus discrete quantity discrimination in dune snail (Mollusca: Gastropoda) seeking thermal refuges. Scientific Reports.

[CR10] Brannon EM, Merritt DJ, Dehaene S, Brannon EM (2011). Evolutionary foundations of the approximate number system. *Space, time and number in the brain*.

[CR11] Butterworth B (2010). Foundational numerical capacities and the origins of dyscalculia. Trends in Cognitive Sciences.

[CR12] Clarke S, Beck J (2021). The number sense represents (rational) numbers. The Behavioral and Brain Sciences.

[CR13] Clayton S, Gilmore C, Inglis M (2015). Dot comparison stimuli are not all alike: The effect of different visual controls on ANS measurement. Acta Psychologica.

[CR14] Cohen P, Cohen P, West SG, Aiken LS (2014). *Applied multiple regression/correlation analysis for the behavioral sciences*.

[CR15] Craig JC, Lyle KB (2001). A comparison of tactile spatial sensitivity on the palm and fingerpad. Perception & Psychophysics.

[CR16] Dehaene, S. (2011). *The number sense: How the mind creates mathematics* (Rev. and updated ed). Oxford University Press.

[CR17] Dehaene S, Changeux J-P (1993). Development of elementary numerical abilities: A neuronal model. Journal of Cognitive Neuroscience.

[CR18] DeWind NK, Brannon EM (2016). Significant Inter-Test Reliability across Approximate Number System Assessments. Frontiers in Psychology.

[CR19] DeWind NK, Adams GK, Platt ML, Brannon EM (2015). Modeling the approximate number system to quantify the contribution of visual stimulus features. Cognition.

[CR20] Dietrich JF, Huber S, Nuerk H-C (2015). Methodological aspects to be considered when measuring the approximate number system (ANS)—A research review. Frontiers in Psychology.

[CR21] Dietrich JF, Nuerk H-C, Klein E, Moeller K, Huber S (2019). Set size influences the relationship between ANS acuity and math performance: A result of different strategies?. Psychological Research.

[CR22] Ernst MO, Banks MS (2002). Humans integrate visual and haptic information in a statistically optimal fashion. Nature.

[CR23] Faul F, Erdfelder E, Buchner A, Lang A-G (2009). Statistical power analyses using G*Power 3.1: Tests for correlation and regression analyses. Behavior Research Methods.

[CR24] Feigenson L, Dehaene S, Spelke E (2004). Core systems of number. Trends in Cognitive Sciences.

[CR25] Gallace A, Tan HZ, Spence C (2007). Multisensory numerosity judgments for visual and tactile stimuli. Perception & Psychophysics.

[CR26] Gebuis T, Reynvoet B (2011). Generating nonsymbolic number stimuli. Behavior Research Methods.

[CR27] Gebuis T, Cohen Kadosh R, Gevers W (2016). Sensory-integration system rather than approximate number system underlies numerosity processing: A critical review. Acta Psychologica.

[CR28] Geisser S, Greenhouse SW (1958). An extension of Box’s results on the use of the * F * distribution in multivariate analysis. The Annals of Mathematical Statistics.

[CR29] Gevers W, Kadosh RC, Gebuis T, Henik A (2016). Sensory integration theory: An alternative to the approximate number system. *Continuous issues in numerical cognition*.

[CR30] Gilmore CK, McCarthy SE, Spelke ES (2010). Non-symbolic arithmetic abilities and mathematics achievement in the first year of formal schooling. Cognition.

[CR31] Gilmore C, Cragg L, Hogan G, Inglis M (2016). Congruency effects in dot comparison tasks: Convex hull is more important than dot area. Journal of Cognitive Psychology.

[CR32] Halberda J, Mazzocco MM, Feigenson L (2008). Individual differences in non-verbal number acuity correlate with maths achievement. Nature.

[CR33] Hendryckx C, Guillaume M, Beuel A, Van Rinsveld A, Content A (2021). Mutual influences between numerical and nonnumerical quantities in comparison tasks. Quarterly Journal of Experimental Psychology.

[CR34] Hosmer DW, Lemesbow S (1980). Goodness of fit tests for the multiple logistic regression model. Communications in Statistics—Theory and Methods.

[CR35] Hyde DC (2011). Two systems of non-symbolic numerical cognition. Frontiers in Human Neuroscience.

[CR36] Izard V, Sann C, Spelke ES, Streri A (2009). Newborn infants perceive abstract numbers. Proceedings of the National Academy of Sciences.

[CR37] Leibovich T, Katzin N, Harel M, Henik A (2017). From “sense of number” to “sense of magnitude”: The role of continuous magnitudes in numerical cognition. Behavioral and Brain Sciences.

[CR38] Leibovich T, Katzin N, Salti M, Henik A (2017). Toward an integrative approach to numerical cognition. Behavioral and Brain Sciences.

[CR39] Lourenco, S. F., & Aulet, L. S. (2022). A theory of perceptual number encoding. *Psychological Review*. Advance online publication. 10.1037/rev0000380 10.1037/rev0000380 35834184

[CR40] Marinova M, Fedele M, Reynvoet B (2021). Weighted numbers. The Behavioral and Brain Sciences.

[CR41] Mou Y, vanMarle K (2014). Two core systems of numerical representation in infants. Developmental Review.

[CR42] Nieder A (2016). The neuronal code for number. Nature Reviews Neuroscience.

[CR43] Olsson L, Östergren R, Träff U (2016). Developmental dyscalculia: A deficit in the approximate number system or an access deficit?. Cognitive Development.

[CR44] Park J, Brannon EM (2013). Training the Approximate number system improves math proficiency. Psychological Science.

[CR45] Pomè A, Thompson D, Burr DC, Halberda J (2021). Location- and object-based attention enhance number estimation. Attention, Perception, & Psychophysics.

[CR46] Qu C, DeWind NK, Brannon EM (2022). Increasing entropy reduces perceived numerosity throughout the lifespan. Cognition.

[CR47] R Core Team (2019). *R: A Language and Environment for Statistical Computing*.

[CR48] Roquet A, Lemaire P (2019). Strategy variability in numerosity comparison task: A study in young and older adults. Open Psychology.

[CR49] Spelke ES, Kinzler KD (2007). Core knowledge. Developmental Science.

[CR50] Szucs D, Nobes A, Devine A, Gabriel FC, Gebuis T (2013). Visual stimulus parameters seriously compromise the measurement of approximate number system acuity and comparative effects between adults and children. Frontiers in Psychology.

[CR51] Tokita M, Ishiguchi A (2016). Precision and bias in approximate numerical judgment in auditory, tactile, and cross-modal presentation. Perception.

[CR52] Tokita M, Ashitani Y, Ishiguchi A (2013). Is approximate numerical judgment truly modality-independent? Visual, auditory, and cross-modal comparisons. Attention, Perception, & Psychophysics.

[CR53] Tomlinson RC, DeWind NK, Brannon EM (2020). Number sense biases children’s area judgments. Cognition.

[CR54] Uluç, I., Velenosi, L. A., Schmidt, T. T., & Blankenburg, F. (2020). Parametric representation of tactile numerosity in working memory. *eNeuro, 7*(1). 10.1523/ENEURO.0090-19.2019 10.1523/ENEURO.0090-19.2019 PMC7029184 31919053

[CR55] Utochkin IS, Vostrikov KO (2017). The numerosity and mean size of multiple objects are perceived independently and in parallel. PLoS One.

[CR56] Wahn B, König P (2017). Is attentional resource allocation across sensory modalities task-dependent?. Advances in Cognitive Psychology.

[CR57] Walsh V (2003). A theory of magnitude: common cortical metrics of time, space and quantity. Trends in Cognitive Sciences.

[CR58] World Medical Association (2013). World medical association declaration of Helsinki: Ethical principles for medical research involving human subjects. The Journal of the American Medical Association.

[CR59] Yu RQ, Zhao J (2015). Numerosity perception is distinct from mean or sum perception. Journal of Vision.

[CR60] Zhang Y, Liu T, Chen C, Zhou X (2019). Visual form perception supports approximate number system acuity and arithmetic fluency. Learning and Individual Differences.

[CR61] Ziegler MC, Drewing K (2022). Get in touch with numbers—An approximate number comparison task in the haptic modality. Attention, Perception, & Psychophysics.

[CR62] Zorzi M, Testolin A (2018). An emergentist perspective on the origin of number sense. Philosophical Transactions of the Royal Society of London. Series B, Biological Sciences.

